# Growth-regulating factor 15-mediated vascular cambium differentiation positively regulates wood formation in hybrid poplar (*Populus alba* × *P. glandulosa*)

**DOI:** 10.3389/fpls.2024.1343312

**Published:** 2024-02-15

**Authors:** Houjun Zhou, Xueqin Song, Meng-Zhu Lu

**Affiliations:** ^1^ The Engineering Research Institute of Agriculture and Forestry, Ludong University, Yantai, China; ^2^ State Key Laboratory of Tree Genetics and Breeding, Research Institute of Forestry, Chinese Academy of Forestry, Beijing, China; ^3^ State Key Laboratory of Subtropical Silviculture, College of Forestry and Biotechnology, Zhejiang A&F University, Hangzhou, China

**Keywords:** hybrid poplar, PagGRF15, vascular cambium, division, xylem differentiation

## Abstract

**Introduction:**

Hybrid poplars are industrial trees in China. An understanding of the molecular mechanism underlying wood formation in hybrid poplars is necessary for molecular breeding. Although the division and differentiation of vascular cambial cells is important for secondary growth and wood formation, the regulation of this process is largely unclear.

**Methods:**

In this study, *mPagGRF15* OE and *PagGRF15-SRDX* transgenic poplars were generated to investigate the function of *PagGRF15*. RNA-seq and qRT-PCR were conducted to analyze genome-wide gene expression, while ChIP‒seq and ChIP-PCR were used to identified the downstream genes regulated by *PagGRF15*.

**Results and discussion:**

We report that *PagGRF15* from hybrid poplar (*Populus alba* × *P. glandulosa*), a growth-regulating factor, plays a critical role in the regulation of vascular cambium activity. *PagGRF15* was expressed predominantly in the cambial zone of vascular tissue. Overexpression of *mPagGRF15* (the mutated version of *GRF15* in the *miR396* target sequence) in Populus led to decreased plant height and internode number. Further stem cross sections showed that the *mPagGRF15* OE plants exhibited significant changes in vascular pattern with an increase in xylem and a reduction in phloem. In addition, cambium cell files were decreased in the *mPagGRF15* OE plants. However, dominant suppression of the downstream genes of *PagGRF15* using *PagGRF15-SRDX* showed an opposite phenotype. Based on the RNA-seq and ChIP-seq results, combining qRT-PCR and ChIP-PCR analysis, candidate genes, such as *WOX4b*, *PXY* and *GID1.3*, were obtained and found to be mainly involved in cambial activity and xylem differentiation. Accordingly, we speculated that *PagGRF15* functions as a positive regulator mediating xylem differentiation by repressing the expression of the *WOX4a* and *PXY* genes to set the pace of cambial activity. In contrast, *PagGRF15* mediated the GA signaling pathway by upregulating *GID1.3* expression to stimulate xylem differentiation. This study provides valuable information for further studies on vascular cambium differentiation mechanisms and genetic improvement of the specific gravity of wood in hybrid poplars.

## 1 Introduction

As one of the fastest-growing industrial trees, hybrid poplars are suitable for cultivation (Sannigrahi et al., 2010). Their widespread distribution also makes them important contributors to ecological diversity. Annotating the poplar genome has provided a way to breed new clones optimized for timber production (Sannigrahi et al., 2010) and has made poplars model plants for tree molecular biology and biotechnology (Brunner et al., 2004). Changes in the cambium during growth, water availability, insect and bacterial attacks, gravitropic effects, and other environmental influences are key energy-related characteristics of lignocellulosic feedstock and can be enhanced by genetic modifications (Sannigrahi et al., 2010). The specific gravity of wood and its lignin and cellulose contents have been proposed as prime targets for genetic modification (Dinus, 2001; Sannigrahi et al., 2010).

Cell proliferation of the vascular cambium generates wood (Smetana et al., 2019). The vascular cambium is a lateral meristem responsible for the production of secondary xylem (wood) inward and secondary phloem outward during secondary growth (Włoch et al., 2023). The differentiation of vascular cambium to xylem and phloem occurs underlying a series of highly ordered and complicated developmental process, which involves initiation, patterning and direction of vascular tissue differentiation (Johnsson and Fischer, 2016). Thus, the pace of the division and differentiation of vascular cambial cells is a rate-limiting step for wood formation. However, the underlying molecular mechanism that regulates vascular cambium expansion and xylem differentiation is not well understood.

Growth-regulating factors (GRFs) are plant-specific transcription factors that are involved in various developmental events and mainly function in organ size control (Hepworth and Lenhard, 2014; Kim, 2019). GRFs contain conserved QLQ and WRC domains in their N-terminal regions, which were the most distinctive characteristics of the family members in plant species (Kuijt et al., 2014). The QLQ domain mediates protein-protein interaction, whereas the WRC domain is necessary for DNA-binding (Omidbakhshfard et al., 2015). Currently, the GRF family consists of 9 genes in *Arabidopsis thaliana* (Kim et al., 2003) and 19 genes in *Populus trichocarpa* (Cao et al., 2016). Most GRFs are the target of miR396, and they work with miR396 to regulate the development of leaves, roots, stems, flowers and seeds (Kim and Tsukaya, 2015; Omidbakhshfard et al., 2015; Kim, 2019). Notably, the function of GRFs in meristem development has increasingly attracted attention of the researchers. Previous studies showed that grf1/2/3/4 displays a shoot meristemless phenotype (Kim and Lee, 2006), but the root apical meristem (RAM) size was enlarged in *grf1/2/3 Arabidopsis*, and the miR396-GRF-PLETHORA (PLT) module was required for the transition of stem cells into transit-amplifying cells to establish the boundary between the stem cell niche and the transit-amplifying region (Rodriguez et al., 2015). GRF directly binds to the promotor of KNOX, a key regulator involved in meristem activity (Kuijt et al., 2014). In addition, GRF-interacting factor 1 (GIF1) can interact with GRFs to regulate meristem determinacy in maize (Zhang et al., 2018). These results suggest that GRFs play an essential role in apical meristem activity by interacting with other regulators. In hybrid poplar 84K (*Populus alba* × *P. glandulosa*), we previously reported that PagGRF12a interacted with PagGIF1b to control *PagXND1a* expression and thereby inhibited xylem development (Wang et al., 2021). Recently, a novel module for *GRF15* regulating secondary vascular development was reported in *P. tomentosa* (Wang et al., 2023). The *PtoTCP20*-miR396d-*PtoGRF15* module mediated vascular development during the transition from primary to secondary vascular development. *PtoTCP20* activated the transcription of miR396d to downregulate *PtoGRF15* expression. *35S:PtoGRF15* plants exhibited delayed secondary growth, indicating that GRF15 was a negative regulator of secondary vascular development in *P. tomentosa* (Wang et al., 2023).

In a previous study, we reported that *PagGRF15* is highly expressed in leaves and can modulate leaf development by regulating expansin genes (Zhou et al., 2019). Overexpression of *PagGRF15* caused significantly enlarged leaves and increased palisade cell size in *mPagGRF15* OE plants, whereas *PagGRF15-SRDX* plants exhibited significantly decreased leaf size and palisade cell area. *PagGRF15-SRDX* plants have opposite effects to the overexpressing lines, because expression of genes downstream of *PagGRF15* would be repressed by SRDX, an active transcriptional repression domain. Moreover, *PagGRF15* exhibits the highest expression in stems among *PagGRFs* (Zhou et al., 2019), implying that *PagGRF15* may play a role in the regulation of vascular cambium activity, which is worth further investigation. In this study, we investigated the role of *PagGRF15* in stem development. Overexpression of *PagGRF15* in *Populus* resulted in increased xylem production and decreased phloem tissue growth, while *PagGRF15-*dominant repressed transgenic plants demonstrated the opposite phenotype. The genes involved in vascular cambium differentiation are targeted by *PagGRF15*. These findings increase the understanding of vascular differentiation mechanisms in hybrid poplar 84K and provide information to support improvements in hybrid poplar cultivation and the specific gravity of wood by genetic modification.

## 2 Materials and methods

### 2.1 Plant materials

Hybrid poplar 84K was used for gene cloning and genetic transformation. *mPagGRF15*OE plants, *PagGRF15pro::GUS* plants and *PagGRF15* SRDX plants were obtained as previously reported (Zhou et al., 2019). All plants were cultivated in a phytotron with a light and dark cycle of 16 h and 8 h at 22°C-25°C.

### 2.2 GUS staining

GUS staining was performed as previously reported (Zhou et al., 2019). Briefly, 1-month-old *PagGRF15pro:GUS* plants were incubated in 90% acetone (v/v) for at least 2 h on ice and then incubated in GUS staining solution (50 mM sodium phosphate, 2 mM potassium ferricyanide, 2 mM potassium ferrocyanide, 0.2% Triton X-100, and 1 mM X-Gluc) for at least 2 h at 37°C. Following staining, plants were cleared with 95% ethanol and preserved in 75% ethanol and photographed. Sections were obtained using a vibratome (VT1000S; Leica, Wetzlar, Germany) at a thickness of 30 μm and photographed using an Axio Imager A1 microscope (Zeiss).

### 2.3 RNA isolation and qRT-PCR

For RNA-seq, samples were collected by scraping the juicy materials from the surface of debarked bark of two-year-old CK and *PagGRF15* transgenic plants grown in the experimental field. Then, total RNA was isolated using the RNeasy Plant Mini Kit (QIAGEN). Six independent cDNA libraries of CK- and *mPagGRF15*-overexpressing plants were sequenced using an Illumina Solexa sequencing platform. Then, RNA-seq reads were aligned to the *P. trichocarpa* genome (Phytozome 10.0). Genes with |log_2_fold change (FC)| > 1 and false discovery rate (FDR) ≤ 0.05 in all three biological replicates were designated as differentially expressed. For qRT-PCR, actin7 (Potri.001G309500, Tang et al., 2019) was used as the internal control.

### 2.4 Microscopy

Different internodes of CK and *PagGRF15* transgenic plants were fixed in 4% paraformaldehyde (prepared with 1×PBS solution, 0.137 M NaCl, 0.0027 M KCl, 0.008 M Na_2_HPO_4_, 0.002 M KH_2_PO_4_, pH 7.2–7.4), dehydrated in a graded ethanol series (30%, 50%, 70%, 90% and 100%), exchanged in a graded Spurr’s resin (ERL 4221:D.E.R. 736:NSA:DMAE=4.10:1.43:5.90:0.10, SPI-CHEM™):ethanol series (1:3, 1:1 and 3:1), and polymerized in 100% Spurr’s resin at 60°C. Then, the samples were sectioned using a microtome (Leica EM UC7) with a thickness of 4 μm, stained with 0.05% (w/v) toluidine blue and visualized using a microscope (OLYMPUS BX51, Olympus).

### 2.5 RNA-seq analysis

For RNA-seq, samples were collected by scraping the juicy materials from the surface of debarked bark of two-year-old CK and *PagGRF15* transgenic plants grown in the experimental field. Then, total RNA was isolated using the RNeasy Plant Mini Kit (QIAGEN). Six independent cDNA libraries of CK- and *mPagGRF15*-overexpressing plants were sequenced using an Illumina Solexa sequencing platform. Then, RNA-seq reads were aligned to the *P. trichocarpa* genome (Phytozome 10.0). Genes with |log_2_fold change (FC)| > 1 and false discovery rate (FDR) ≤ 0.05 in all three biological replicates were designated as differentially expressed (BioProject ID: PRJNA693036).

### 2.6 ChIP-seq and ChIP-qPCR

ChIP assays were performed as described previously (Li et al., 2014). Briefly, 2.5 g stems of 1-month-old *35S:GFP-PagGRF15* plants were collected and ground to a fine powder in liquid nitrogen. The powder was transferred to 20 ml cross-linking buffer for a total of 30 min under vacuum at room temperature. 2.5 ml of 2 M glycine was added to quench the cross-linking reaction, and the solution centrifuged at 1,800g for 10 min. The pellet was resuspended in 30 ml buffer 1 (0.4 M sucrose, 10 mM Tris-HCl (pH 8.0), 5 mM β-mercaptoethanol, 1 mM PMSF, 1 µg/ml pepstatin A and 1 µg/ml leupeptin) and the mixture was gradually passed through three layers of Miracloth into a new, ice-cold 50-ml conical tube, and the Miracloth squeezed to collect all of the liquid. The solution was centrifuged at 1,800g for 10 min at 4°C, the supernatant discarded, and the pellet resuspended in 2 ml buffer 2 (0.25 M sucrose, 10 mM Tris-HCl (pH 8.0), 5 mM β-mercaptoethanol, 10 mM MgCl_2_, 1% (vol/vol) Triton X-100, 1 mM PMSF, 1 µg/ml pepstatin A and 1 µg/ml leupeptin) and transferred to a 2.0-ml microcentrifuge tube. After centrifugation at 16,000g for 10 min at 4°C, the pellet was resuspended in 400 µl of buffer 3 (1.7 M sucrose, 10 mM Tris–HCl (pH 8.0), 5 mM β-mercaptoethanol, 2 mM MgCl_2_, 0.15% (vol/vol) Triton X-100, 1 mM PMSF, 1 µg/ml pepstatin A and 1 µg/ml leupeptin). After centrifugation at 16,000g for 10 min at 4°C, the pellet was resuspended in 400 µl of buffer 3 once more, then centrifuged at 16,000g for 1 h at 4°C and the supernatant removed. The pellet (chromatin) was resuspended in 400 µl of lysis buffer (50 mM Tris-HCl (pH 8.0), 10 mM EDTA, 1% (wt/vol) SDS, 1 mM PMSF, 1 µg/ml pepstatin A and 1 µg/ml leupeptin) and sonicated. Then, the sample was centrifuged for chromatin isolation. Chromatin extracted from *35S:GFP-PagGRF15* plants was immunoprecipitated with anti-GFP antibody (No. 11814460001; Roche) with a Plant ChIP-seq kit (Diagenode). Following decrosslinking, isolation, and purification of the immunoprecipitated DNA, libraries were constructed and subjected to sequencing on the Illumina HiSeq 2000 platform. ChIP-seq reads were aligned to the *Populus trichocarpa* genome (Phytozome 10.0). Peaks were identified as significantly enriched (P < 0.05) in each of the ChIP-seq libraries compared with input DNA. Three independent biological replicates of the ChIP assay were performed. For ChIP-qPCR, SYBR Premix Ex Taq (TaKaRa) was used to quantify DNA targets immunoprecipitated by anti-GFP antibodies relative to input DNA with three biological replicates each with four technical replicates. The primers used are listed in **Supplementary Table 5**. Actin7 (POPTR_0019s02630.1, Li et al., 2014) was used as the internal control, and the fold enrichment of the DNA target was relative to the input sample.

## 3 Results

### 3.1 Expression pattern of *PagGRF15* during vascular development

**Figure 1:**
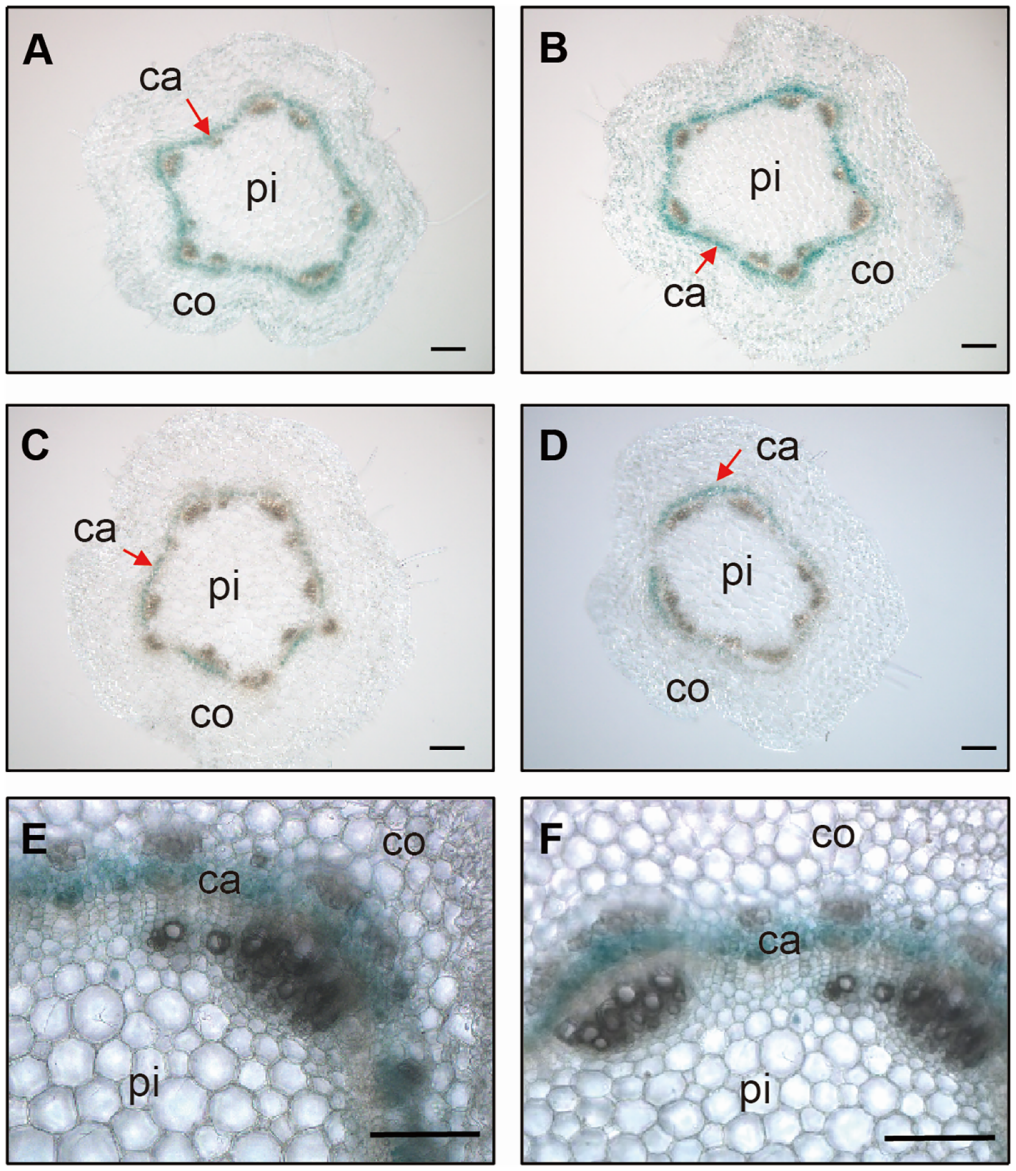
Expression patterns of *GRF15* in stems. **(A-D)** Stem sections of the 2^nd^ **(A)**, 3^rd^ **(B)**, 4^th^ **(C)** and 5^th^ **(D)** internodes from 4-week-old *PagGRF15pro:GUS* transgenic plants. Bars = 100 μm. **(E, F)** Further cross sections of the 2^nd^ **(E)** and 3^rd^ **(F)** internodes showed that GUS signals were concentrated in the vascular cambium. Bars = 100 μm. Ca, cambium; pi, pith; co, cortex.

In a previous study, we reported that *PagGRF15* was the most highly expressed *PagGRF* in stem tissue, with a higher expression in young stems than in mature stems (Zhou et al., 2019). GUS staining assays revealed strong signals in young stems (**Supplementary Figure 1B**), consistent with the expression of *GRF15* from the apex to the 5^th^ internode detected through joint PacBio Iso**-**Seq and RNA**-**seq analysis (**Supplementary Figure 1**; Chao et al., 2019). To examine the expression pattern of *PagGRF15* in detail, we analyzed cross sections of 1-month-old *PagGRF15pro:GUS* transgenic seedlings. Cross sections of the 2^nd^ to 5^th^ internodes revealed strong signals in the vascular tissue and were emphasized in the cambial zone (**Figure 1**). qRT-PCR results also confirmed the expression of *PagGRF15* in vascular tissue (**Supplementary Figure 1C**). These results suggest that *PagGRF15* plays a crucial role in vascular development, likely through the regulation of cambium activity.

### 3.2 Altered *PagGRF15* expression led to the alteration of secondary vascular development

**Figure 2:**
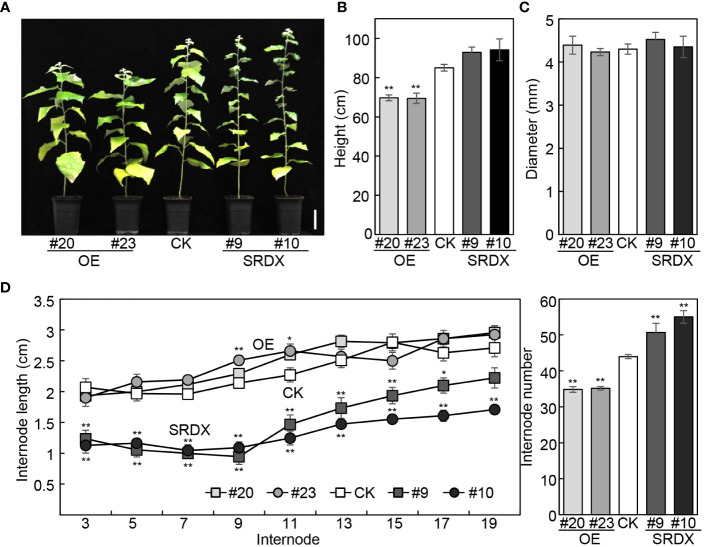
Growth characteristics of *mPagGRF15* overexpression (OE) and *PagGRF15* dominant repression (SRDX) transgenic plants. **(A)** Photograph of 4-month-old *mPagGRF15* OE and *PagGRF15-SRDX* transgenic plants. Bar = 10 cm. **(B, C)** Plant height **(B)** and basal diameter **(C)** of 4-month-old *mPagGRF15* OE and *PagGRF15-SRDX* transgenic plants. **(D)** Internode length and internode number of 4-month-old mPagGRF15 OE and internode number of 4-month-old *mPagGRF15* OE and *PagGRF15-SRDX* transgenic plants. Data are presented as the means ± SDs (n = 6). Asterisks indicate significant differences (Dunnett test) compared to CK. CK, the control line. *, *P* ≤ 0.05; **, *P* ≤ 0.01.

We have reported that the height of *mPagGRF15* OE plants was decreased, while *PagGRF15-SRDX* grew slightly higher than the nontransgenic control (CK) plants (Zhou et al., 2019). Statistically, 4-month-old *mPagGRF15* OE transgenic plants demonstrated an approximately 16.5% decrease in plant height, while *PagGRF15-SRDX* plants displayed an approximately 10% increase (**Figures 2A, B**). The basal diameter, however, was not significantly altered in either *PagGRF15* transgenic plant (**Figure 2C**). We also counted the internode number and measured the internode length of all transgenic lines. The internode number was decreased by 20% in *mPagGRF15* OE transgenic plants but increased by 20% in *PagGRF15-SRDX* plants. The internode length showed no obvious alteration in *mPagGRF15* OE transgenic plants but decreased in *PagGRF15-SRDX* plants compared with CK plants (**Figure 2D**). Interestingly, the internode length showed no significant difference in *mPagGRF15* OE plants but decreased in *PagGRF15-SRDX* plants (**Figure 2D**). These results indicated that PagGRF15 could affect stem development by regulating the differentiation and elongation of internodes.

**Figure 3:**
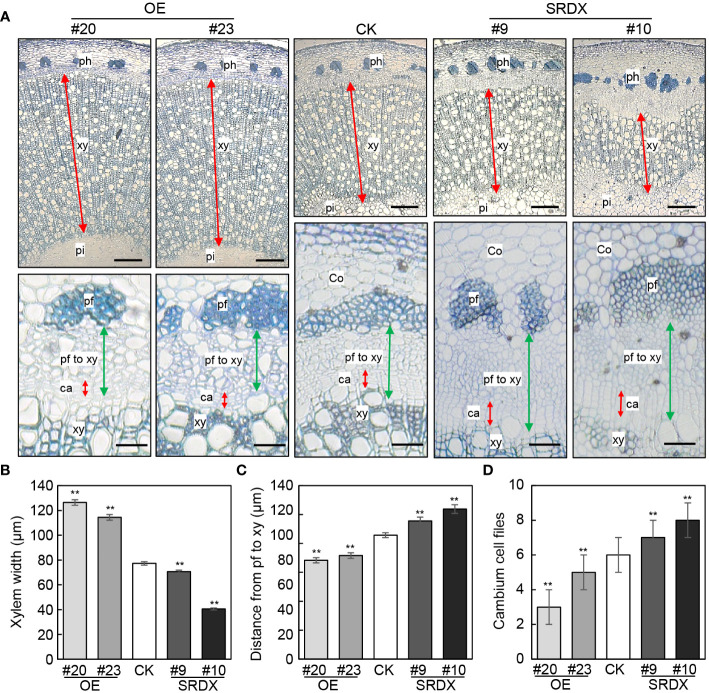
Stem development of *mPagGRF15* OE and *PagGRF15-SRDX* transgenic plants. **(A)** Cross sections of the 9^th^ internode from 4-month-old *mPagGRF15* OE and *PagGRF15-SRDX* plants. Co, cortex; ph, phloem; pf, phloem fiber; xy, xylem; ca, cambium. Bar = 200 µm in the upper panel and bar = 20 µm in the lower panel. **(B-D)** Xylem width, phloem width and cambium cell files of the 9^th^ internode from 4-month-old *mPagGRF15* OE and *PagGRF15-SRDX* transgenic plants, respectively. Data are presented as the means ± SDs. 3 separate plants and 20 biological replicates were used for the statistical analyses in **(B-D)**. Asterisks indicate significant differences (*t* test) compared to CK. CK, the control line. **, *P* ≤ 0.01.

We then assessed the vascular tissues in *PagGRF15* transgenic plants in comparison with CK plants. Cross sections of the 1^st^ to 6^th^ internodes of CK and *PagGRF15* transgenic plants were obtained, and the vascular cambium ring was not significantly different between *PagGRF15* transgenic and CK plants (**Supplementary Figure 2**). Subsequently, cross sections of 4-month-old *PagGRF15* transgenic and CK plants were analyzed. Cross sections of the 7^th^, 9^th^ and 23^rd^ internodes of CK and *PagGRF15* transgenic plants were obtained, and secondary xylem developed faster in *mPagGRF15* OE plants but slower in *PagGRF15-SRDX* plants than in CK plants (**Figures 3** and **Supplementary Figure 3**). Statistical data showed that the width of the secondary xylem from *mPagGRF15* OE transgenic plants was increased compared with that of CK plants, with an approximately 55% increase in the 9^th^ internode (**Figures 3A, B**). In contrast, *PagGRF15*-*SRDX* plants exhibited a narrower xylem zone, with an average of a 28% decrease in the 9^th^ internode (**Figures 3A, B**). On the other hand, the distance from phloem fiber to xylem in the 9^th^ internode decreased by approximately 28% on average in the *mPagGRF15* OE plants but increased by 17% in the *PagGRF15-SRDX* plants (**Figure 3C**). The number of layers of vascular cambium in the 9^th^ internode of 4-month-old seedlings was significantly increased in *mPagGRF15* OE, and decreased in *PagGRF15-SRDX* as compared with CK plants (**Figure 3D**). These results suggested that *PagGRF15* mediated secondary vascular development by affecting cambial activity to set the pace of xylem and phloem differentiation.

### 3.3 The potential downstream genes of PagGRF15

For determination of how *PagGRF15* regulates vascular cambial differentiation, genome-wide gene expression in the cambial zone of *mPagGRF15* OE plants was profiled by RNA-seq. Analysis of transcript abundance showed that 2013 genes were upregulated and 1487 genes were downregulated in the *PagGRF15* OE transgenic plants (**Supplementary Table 1**). Gene Ontology (GO) enrichment analysis indicated that the differentially expressed genes (DEGs) were associated with xylem development, glucuronoxylan metabolic process, and xylem biosynthetic process (**Supplementary Figure 4**), indicating the regulatory role of PagGRF15 in vascular cambial differentiation. To further identify the downstream genes regulated by *PagGRF15*, we performed chromatin immunoprecipitation followed by high-throughput DNA sequencing (ChIP-seq) using GFP-m*PagGRF15* OE plants (**Supplementary Figure 5**). Illumina libraries were sequenced for three independent ChIP-seq biological replicates for GFP antibody libraries, and the DNA ‘input’ libraries were used as a control for ChIP-seq peak calling. A total of 18261 enriched peaks belonging to 13660 genes were identified (**Supplementary Table 2**). Next, the above DEGs found in the transcriptional profile were screened in ChIP-seq data, and 1241 overlapping genes were found (**Supplementary Table 3**). In particular, 81 of these genes were previously reported to be involved in vascular development (**Supplementary Table 4**, Ye and Zhong, 2015; Zhong and Ye, 2015). For instance, *GID1.3* (Mauriat and Moritz, 2009) is involved in regulating xylem differentiation, while *WhOX4b* (Kucukoglu et al., 2017) and *PXY* (Etchells and Turner, 2010) are marker genes for cambial cell activity.

**Figure 4:**
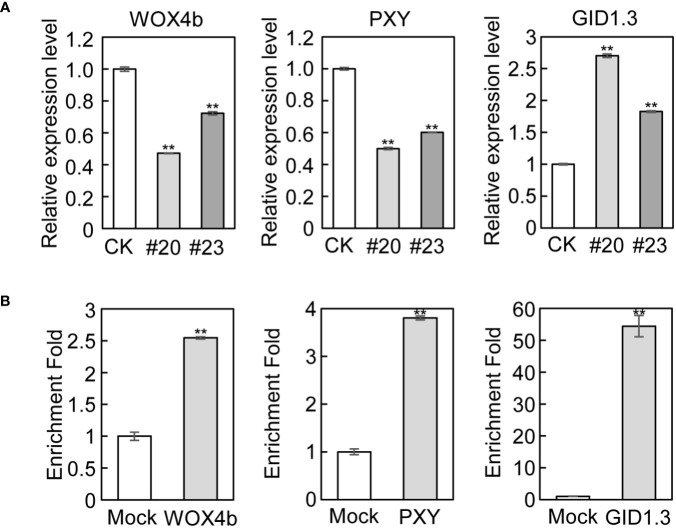
Genes related to vascular development in *mPagGRF15* OE plants. **(A)** qRT-PCR detection of the expression of *WOX4b*, *PXY* and *GID1.3* in *mPagGRF15* OE plants. **(B)** ChIP-qPCR verification of *WOX4b*, *PXY* and *GID1.3* regulated by *PagGRF15.* Data are presented as the means ± SDs. 3 biological and technical replicates were used for the statistical analyses. Asterisks indicate significant differences (*t* test) compared to CK. CK, the control line. **, *P* ≤ 0.01.

To validate the RNA-seq and ChIP-seq results, we performed qRT-PCR and ChIP-qPCR to quantify the above marker genes involved in cambial activity and xylem differentiation. Consistent with the RNA-seq results, the expression of *WOX4b*, *PXY*, and *APL* was downregulated, while the expression of *GID1.3*, *ERF6, JAS1, and MYC2* was upregulated in *mPagGRF15* OE plants in qRT-PCR detection (**Figures 4A** and **Supplementary Figure 6**). Consistent with the ChIP**-**seq results, ChIP-qPCR showed more than 2-fold, 3-fold, and 50-fold enrichment in binding over the input control for *WOX4b*, *PXY*, and *GID1.3*, respectively (**Figure 4B**), suggesting that these genes could be directly regulated by *PagGRF15*.

## 4 Discussion

GRFs are reported to be involved in many developmental events, including stem development (Kim, 2019). Overexpressing *OsGRF1* in *Arabidopsis* completely abolished stem elongation (van der Knaap et al., 2000). Similarly, overexpression of *ZmGRF10* and *ZmGRF1^R^* (miR396a-resistant version of GRF1) in maize both led to a reduction in plant height (Wu et al., 2014; Nelissen et al., 2015). However, overexpressing *OsGRF3* and *OsGRF10* in rice could increase tiller length, while downregulation of the expression of *GRF* resulted in visibly shorter internodes (Kuijt et al., 2014). In hybrid poplar 84K, we reported that plants overexpressing *PagGRF12a* exhibited no significant alteration in plant height (Wang et al., 2021). In addition to *P. tomentosa*, *35S:PtoGRF15* plants also showed no significant difference in height (Wang et al., 2023). Here, we found that overexpression of *PagGRF15* could lead to a decrease in plant height. Thus, different GRFs may play different roles in stem elongation regulation, and *PagGRF15* is a negative regulator of plant height in hybrid poplar 84K. Secondary growth is another aspect of stem development in woody plants. Poplar trees overexpressing *PagGRF12a* as well as *PtoGRF15* showed delayed secondary growth and xylem production reduction (Wang et al., 2021, 2023), indicating the negative regulatory role of GRF in vascular development. We also found that abnormal expression of *PagGRF15* could affect vascular tissue development. Plants with *PagGRF15* overexpression exhibited increased xylem production, whereas *PagGRF15-SRDX* plants showed decreased xylem production. Interestingly, the plant height decreased while the secondary xylem width increased in *mPagGRF15* OE plants, while *PagGRF15-SRDX* plants demonstrated the opposite phenotype. Therefore, *PagGRF15* functions as an activator that regulates not only leaf size but also secondary xylem growth in woody plants.

Previous reports have shown that GRF may have a role in meristem activity (Kim and Lee, 2006; Rodriguez et al., 2015; Zhang et al., 2018), and gene function in the meristem always also has a role in vascular cambium activity (Groover, 2005). In secondary growth, xylem and phloem are produced by cambium activity during wood formation (Spicer and Groover, 2010). Here, we showed that *PagGRF15* is highly expressed in vascular cambium and that its overexpression resulted in fewer cell files in the cambial zone and more cell files in the secondary xylem zone, while *PagGRF15-SRDX* plants had the opposite phenotype, indicating that PagGRF15 could affect secondary xylem development by reducing cambial cell proliferation but accelerating xylem differentiation. *AtGRF5* was reported to accelerate cell proliferation activities and reduce cell expansion (an indication of differentiation in plants) to affect the movement of the cell cycle arrest front (AF) during leaf development (Rodriguez et al., 2010; Vercruyssen et al., 2015). In poplar, we reported that *PagGRF12a* inhibits xylem development by upregulating *PagXND1a* expression (Wang et al., 2021). It was reported that XND1 affects xylem formation by modulating the differentiation of cambial cells to xylem cells (Zhao et al., 2008; Tang et al., 2018; Grant et al., 2010; Zhao et al., 2020). Recently, in *P. tomentosa*, *PtoGRF15* was found to delay secondary vascular development by decreasing the number of layers of xylem cells between primary vascular bundles during the transition from primary to secondary vascular development (Wang et al., 2023). Moreover, *PtoTCP20* integrated with the *miR396d*-*PtoGRF15* regulatory module and thereby formed the *PtoTCP20*-*miR396d*-*PtoGRF15* signaling cascade that functions in secondary vascular development (Wang et al., 2023). In hybrid polar 84K, we showed that PagGRF15 was localized in the vascular cambium zone, which contributed to the development of secondary tissues, indicating the potential roles of PagGRF15 in vascular development during wood formation. Overexpression of *PagGRF15* caused an enlarged xylem width, a decreased amount of phloem and a significant reduction in cambium dividing cells, whereas *PagGRF15-SRDX* plants produced a decreased amount of xylem, an enlarged phloem width and an increased number of cambium dividing cells, resulting in no significant changes in the stem diameter of *PagGRF15* transgenic plants compared with CK plants. These results suggested that PagGRF15 in hybrid poplar acted as a positive regulator of xylem development by affecting cambial activity to set the pace of xylem and phloem. Taken together, the results indicated that GRF15 participates in more complex regulatory loops in secondary vascular tissue growth.

WOX4 is the target of PXY, and both are marker genes for cambial activity (Etchells et al., 2013, 2015; Kucukoglu et al., 2017). PXY is a cambium-specific leucine-rich repeat receptor-like kinase that acts as a receptor of the peptide ligands CLE41/CLE44/TDIF, which stimulates WOX4 transcription factor activity and regulates the proliferation of cambial cells and vascular tissue patterning in stems by interaction with CLE peptides (Sugimoto et al., 2022). WOX4 plays a dominant role in regulating the activity of the vascular cambium (Kucukoglu et al., 2017). In *Arabidopsis*, AtWOX4 is necessary for stimulating the proliferation of procambium/cambium stem cells, but not required for repressing their commitment to become xylem cells (Hirakawa et al., 2010), whereas PttWOX4 not only promotes cambial proliferation, but also enhances the differentiation of secondary xylem in poplar (Kucukoglu et al., 2017). Repressed *PttWOX4a* expression could result in reduced vascular cambium width (Kucukoglu et al., 2017), while overexpression of *PttPXY* could result in increased cambial cell division (Etchells et al., 2015). In *P. tomentosa*, PtoTCP has reported to interact with PtoWOX4a to control vascular cambium proliferation and promote xylem cell differentiation by activating *PtoWND6* transcription (Hou et al., 2020). Recently, the genetic regulatory network underlying WOX4-mediated wood formation at the post-transcriptional level has been elucidated (Tang et al., 2022). *PagWOX4 RNAi* transgenic plants exhibited severely inhibited secondary growth with a reduced proportion of xylem and significantly reduced layers of cambial cells. Genetic analysis showed that PagDA1 functions as a repressor to destabilize PagWOX4 mediated by PagDA2 to affect cambium development (Tang et al., 2022). As shown in this study, the expression of *PagWOX4b* and *PagPXY* was downregulated in *mPagGRF15* OE plants and resulted in decreased cambial cell files. In addition, PagGRF15 can directly regulate *PagWOX4b* and *PagPXY*, as revealed by ChIP-seq and ChIP-qRT analysis. Therefore, GRF15 may be involved in the PXY-WOX pathway to set the pace of cambial activity. Interestingly, *mPagGRF15* OE plants exhibited a significantly increased proportion of xylem, suggesting that there may be another regulatory network underlying PagGRF15-mediated wood formation.

GID is a soluble GA receptor that functions as a key mediator of GA response pathways (Mauriat and Moritz, 2009). The GA/GID1 complex binds to DELLA and leads to the degradation of DELLA to derepress GA signaling (Mauriat and Moritz, 2009). In hybrid aspen, the pith/xylem ratio was lower in *PttGID1.3*-overexpressing plants than in wild-type plants, indicating that overexpression of *PttGID1.3* stimulates xylem differentiation (Mauriat and Moritz, 2009). The xylem width was significantly increased in *mPagGRF15* OE plants compared to CK plants (**Figures 3** and **Supplementary Figure 4**). Thus, the upregulated *PagGID1.3* could be responsible for the enlarged xylem observed in *mPagGRF15* OE plants.

## 5 Conclusions

Hybrid poplars are among the fastest-growing industrial trees in China. *P. alba* × *P. glandulosa* is a hybrid poplar species of economic and ecological importance. The specific gravity of wood has been proposed as a prime target for genetic modification. Accordingly, functional analysis of PagGRF15 from *P. alba* × *P. glandulosa* was performed to improve the understanding of the molecular mechanisms of wood formation. Overexpression of *PagGRF15* in hybrid poplar positively promoted xylem differentiation during plant secondary growth. RNA-seq and ChIP-seq results combining qRT-PCR and ChIP-PCR analysis revealed that PagGRF15 may be involved in setting the pace of cambial activity by downregulating WOX4b and PXY expression and mediating the GA signaling pathway by accelerating *PagGID1.3* expression to stimulate xylem differentiation. The results provide valuable information for further studies on the wood formation mechanisms of hybrid poplar with the aim of improving the specific gravity of wood.

## Data availability statement

The original contributions presented in the study are publicly available. This data can be found here: https://www.ncbi.nlm.nih.gov/bioproject/; PRJNA693036.

## Author contributions

HZ: Data curation, Formal analysis, Funding acquisition, Investigation, Validation, Writing – original draft, Writing – review & editing. XS: Conceptualization, Formal analysis, Funding acquisition, Methodology, Project administration, Visualization, Writing – original draft, Writing – review & editing. ML: Conceptualization, Project administration, Supervision, Writing – review & editing, Methodology.
