# Supplementary material for: Growth-regulating factor 15-mediated vascular cambium differentiation positively regulates wood formation in hybrid poplar (Populus alba × P. glandulosa)
Source: Front Plant Sci. 2024 Feb 15;15:1343312. doi: 10.3389/fpls.2024.1343312 (PMC10902170; doi:10.3389/fpls.2024.1343312)
Supplement: Supplementary file 1 [file DataSheet_1.docx]

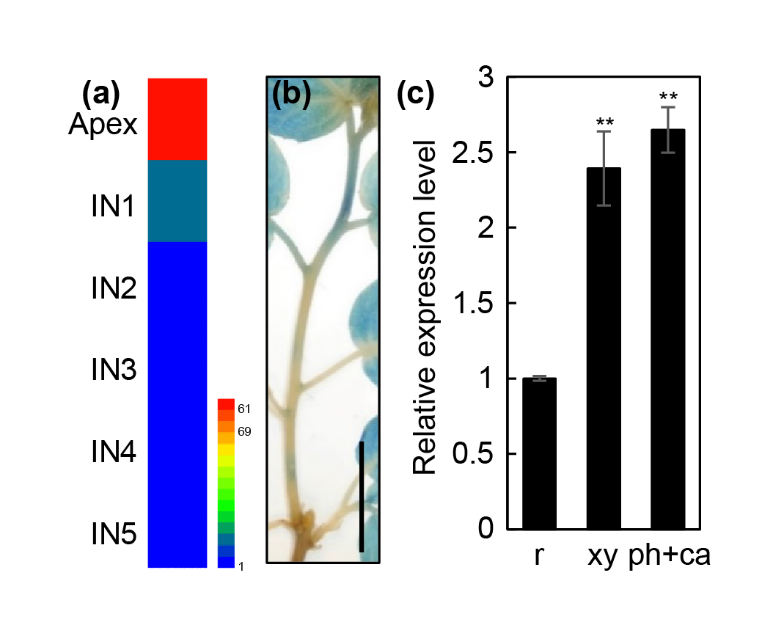


**Figure S1.** Expression profile of *PagGRF15*. (a) Expression of *GRF15* from the apex to the 5^th^ internode (Chao et al., 2019). (b) GUS signals were observed in stems. Bar = 1 cm. (c) qRT‒PCR detection of *PagGRF15* transcript abundance in roots (r), developing xylem (xy), and phloem + cambium (ph + ca). The expression level of *PagGRF15* in root tissues was set at 1 and used to determine the relative abundance for the other samples. *Actin* (Potri.001G309500) was used as an internal control. Data are presented as the means ± SDs (n = 6). Asterisks indicate significant differences (*t* test) compared to CK. CK, the control line. *, *P* ≤ 0.05; **, *P* ≤ 0.01.


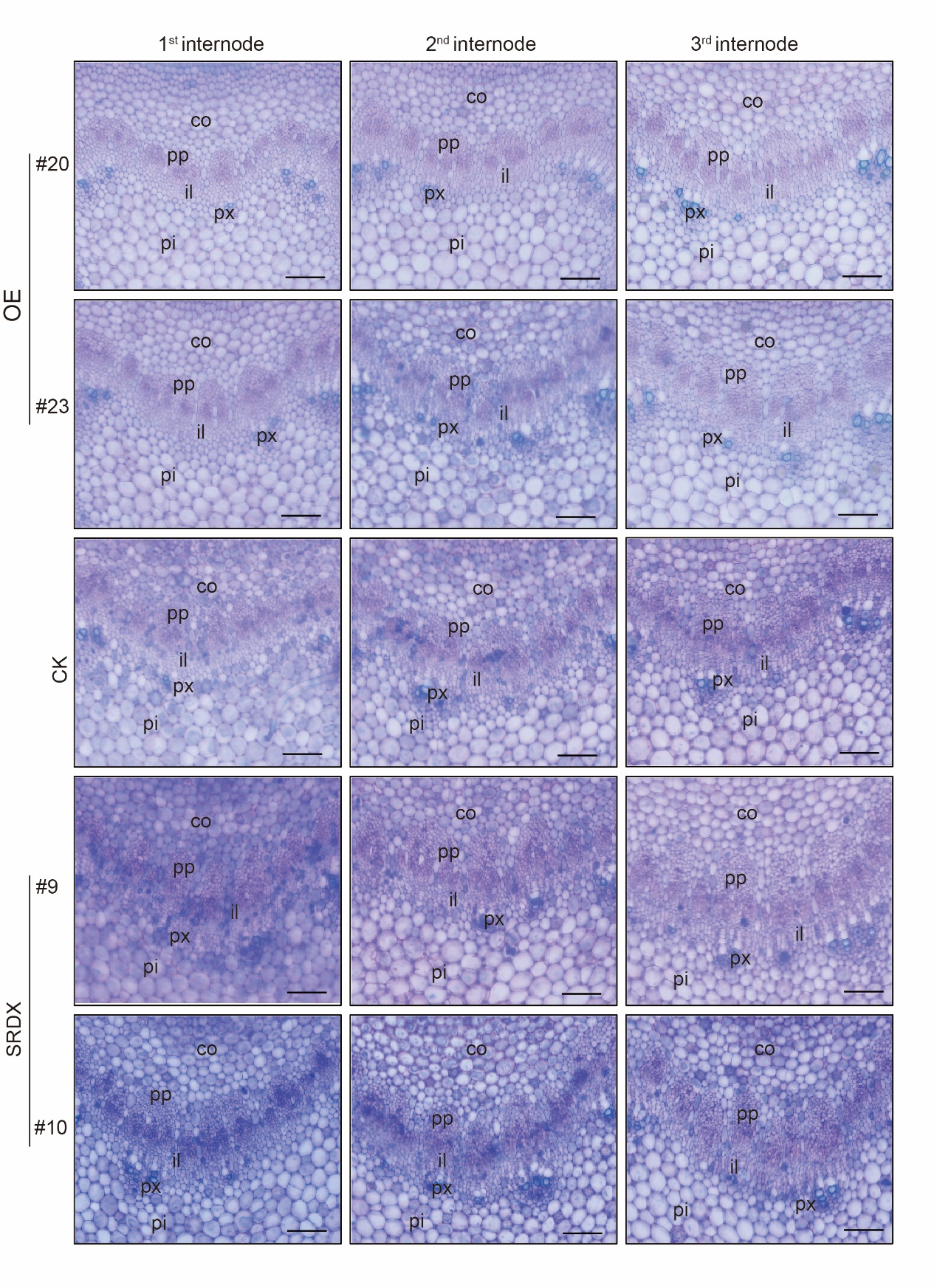

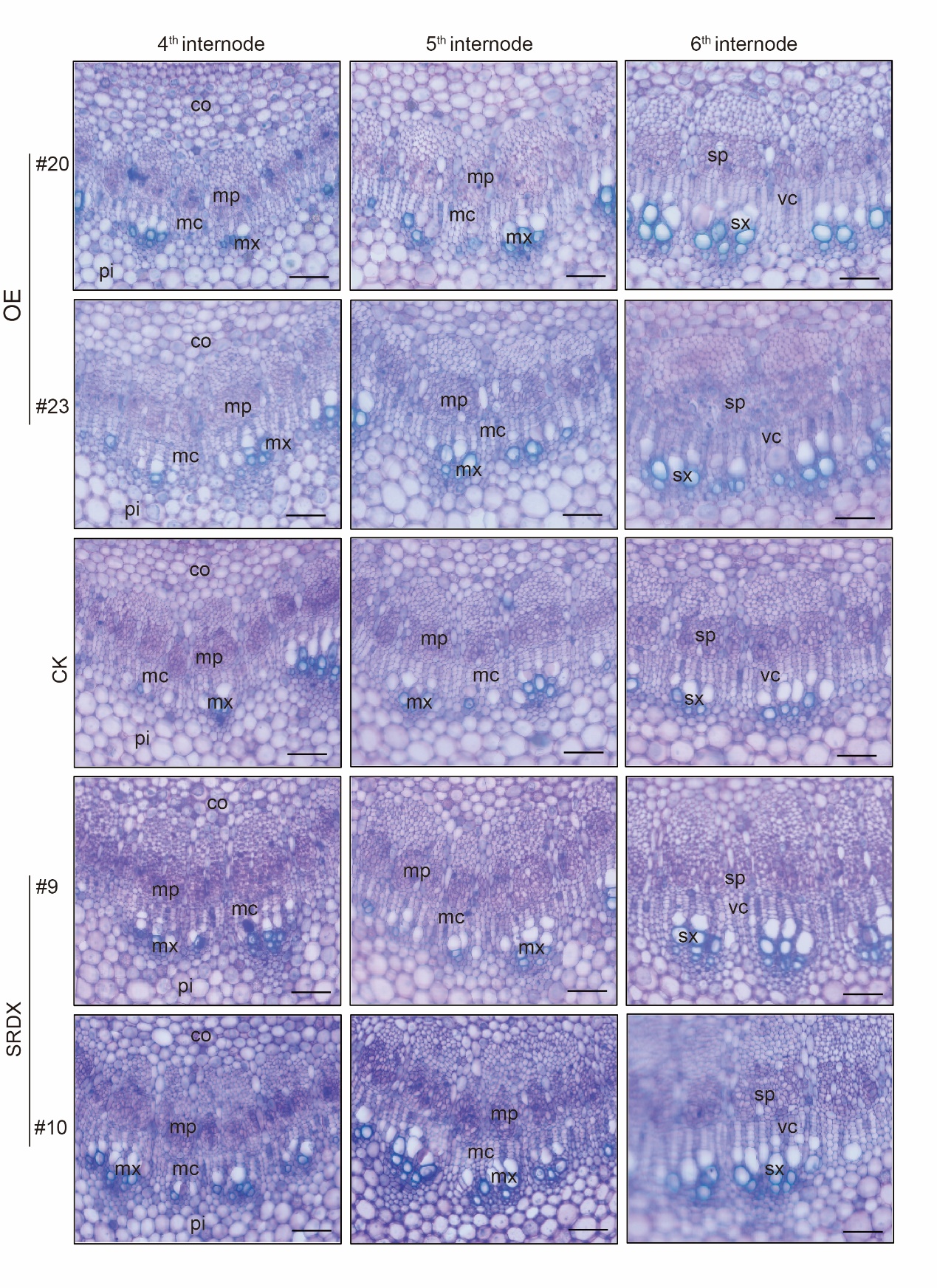


**Figure S2.** Cross sections of the 1^st^ to 6^th^ internodes of 2-month-old CK, *mPagGRF15* OE and *PagGRF15-SRDX* transgenic poplars. Pp, protophloem; px, protoxylem; pc, procambium; il, initiating layer; mx, metaxylem; mp, metaphloem; mc, metacambium; vc, vascular cambium; sp, secondary phloem; sx, secondary xylem. The procambium-cambium meristematic continuum can be subdivided into the procambium, initiating layer, metacambium and cambium (Hou et al., 2020). CK, the control line. 3 separate plants and 20 biological replicates were used for the statistical analyses. Bar = 100 µm.


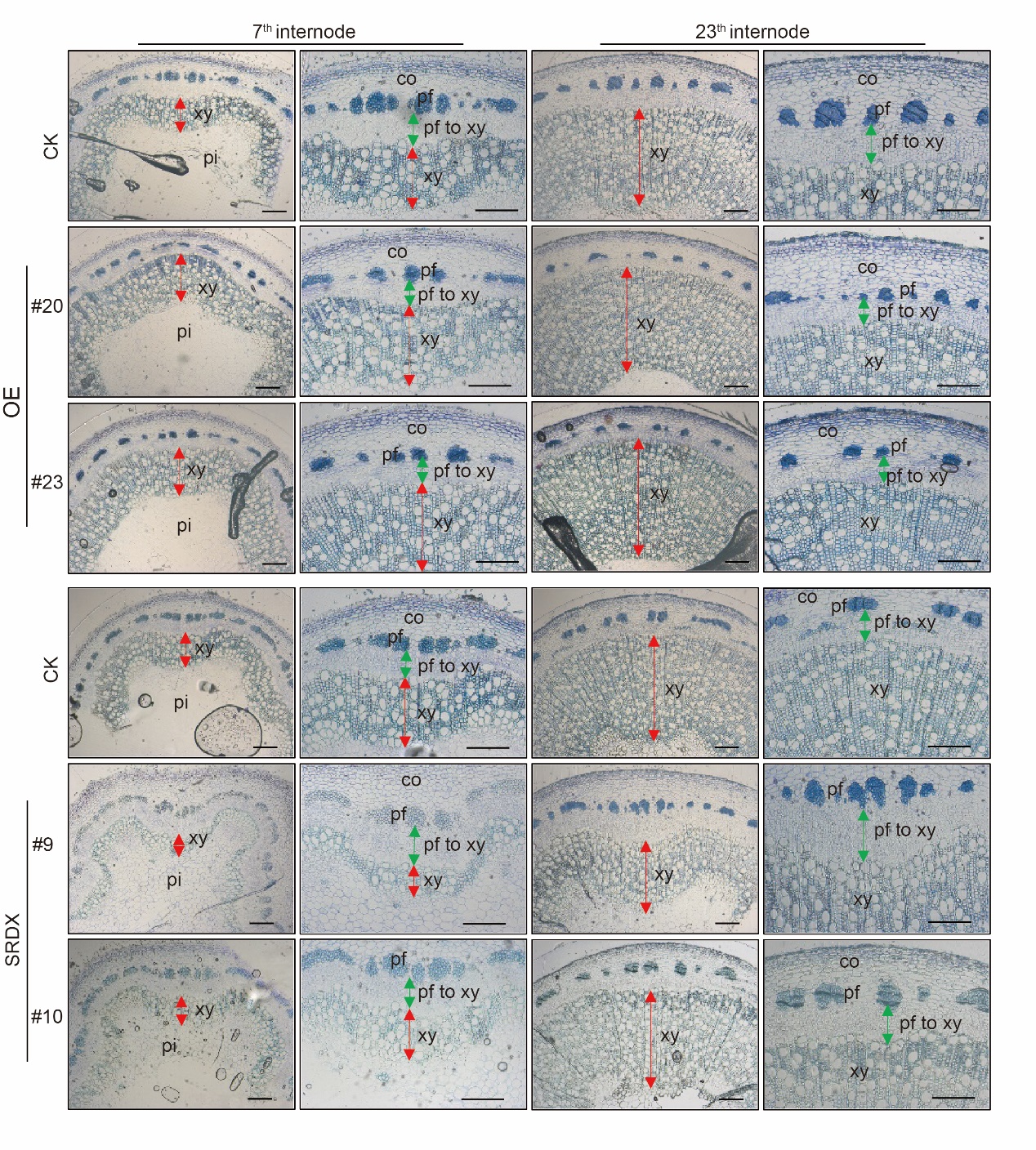


**Figure S3.** Cross sections of the 7^th^ and 23^th^ internodes of 4-month-old CK, *mPagGRF15* OE and *mPagGRF15-SRDX* transgenic poplars. Co, cortex; ph, phloem; pf, phloem fiber; xy, xylem; ca, cambium. CK, the control line. 3 separate plants and 20 biological replicates were used for the statistical analyses. Bar = 100 µm.


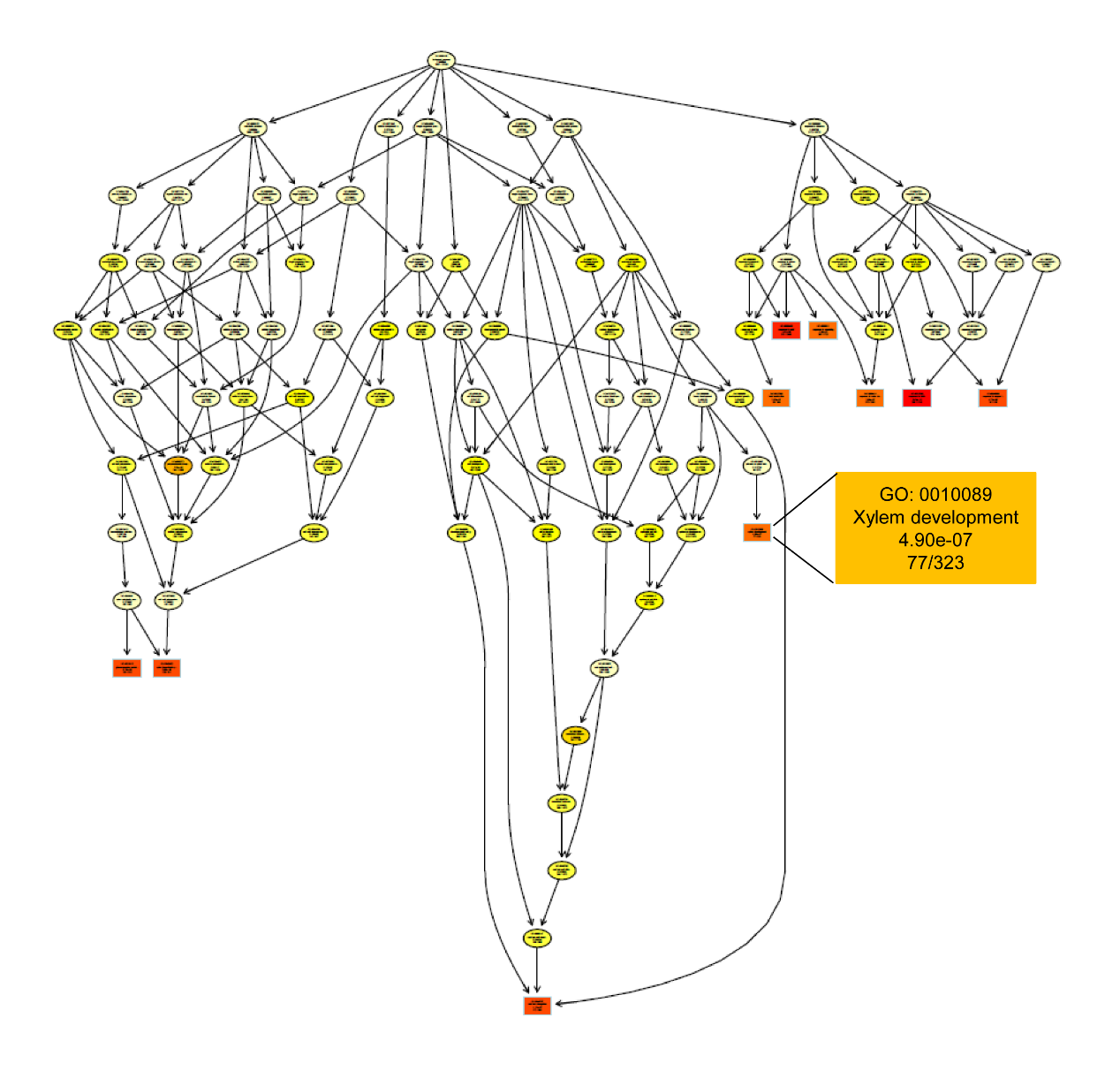


**Figure S4.** GO enrichment of differentially expressed genes. GO: 0010089 (xylem development) was one of the ten top GO terms for GO enrichment of biological process.


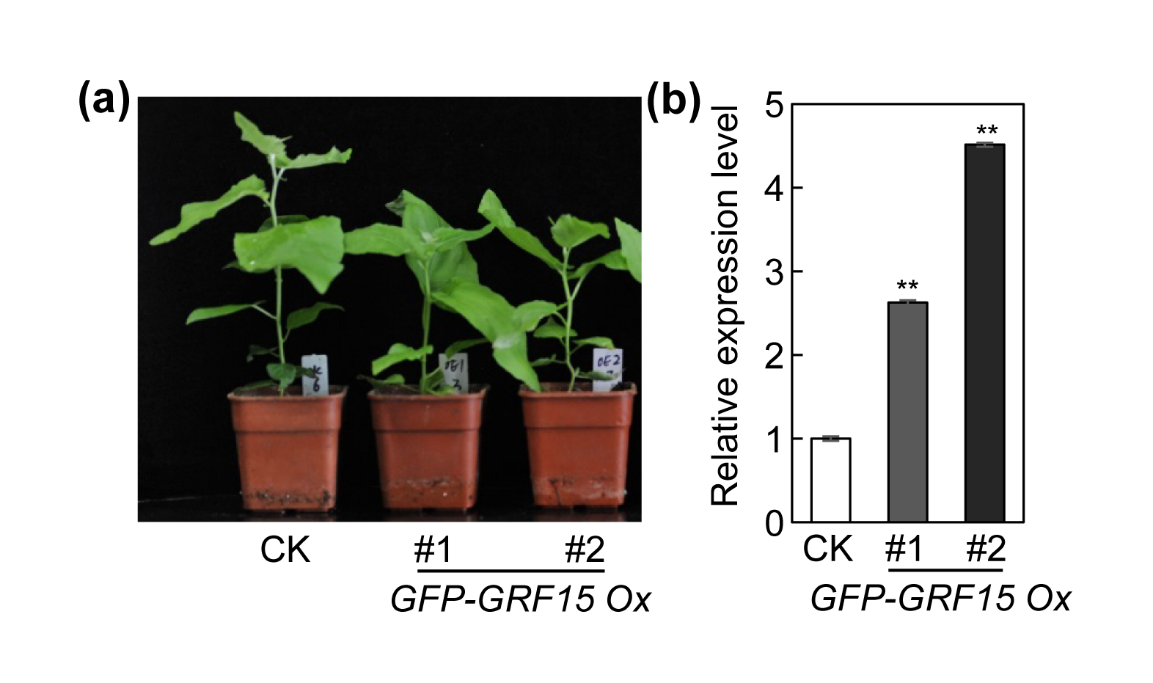


**Figure S5.** GFP-*mPagGRF15* OE transgenic poplars. (a) Photograph of one-month-old plants. (b) Expression level of *PagGRF15* in OE transgenic plants. Data are presented as the means ± SDs of 3 biological and technical replicates. Asterisks indicate significant differences (*t* test) compared to CK. CK, the control line. **, *P* ≤ 0.01.


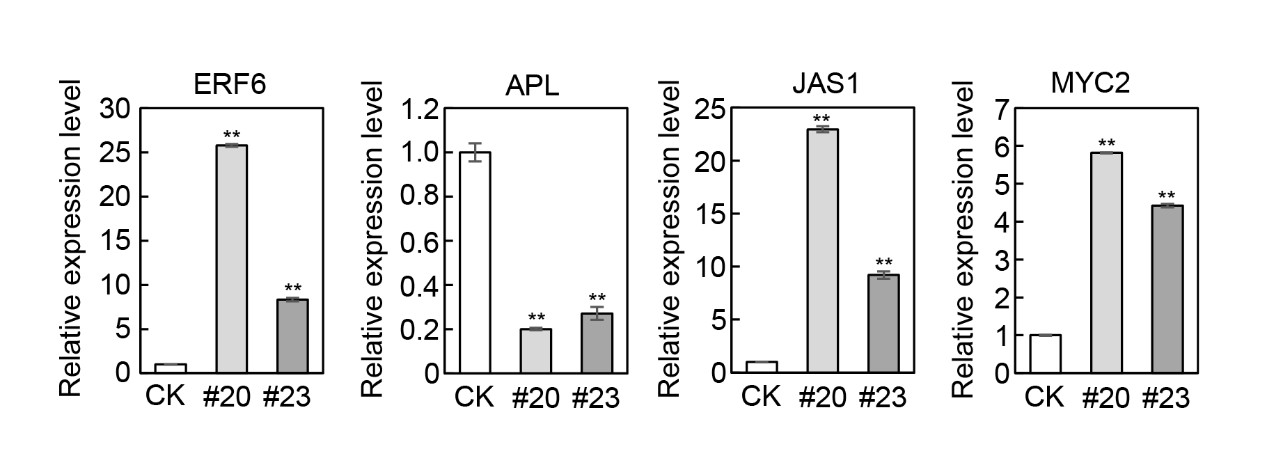


**Figure S6.** Genes characterized in CK and *mPagGRF15* OE plants. qRT‒PCR detection of the expression of genes related to cambial cell differentiation. Data are presented as the means ± SDs of 3 biological and technical replicates. Asterisks indicate significant differences (*t* test) compared to CK. CK, the control line. **, *P* ≤ 0.01.
